# Some Practical Points Involved in the Relation of the Upper to the Lower Teeth

**Published:** 1891-02

**Authors:** 


ARTICLE VI.

### SOME PRACTICAL POINTS INVOLVED IN THE RELATION OF THE UPPER TO THE LOWER TEETH.

---

Mr. Matheson grouped his remarks under the following heads:—(1.) The articulation of the teeth in relation to the etiology and treatment of approximal decay. (2.) Irregularities of articulation as productive of pathological conditions of the alveolo-dental periosteum. (3.)

The "bite," as affecting certain modes of treatment adopted in the correction of irregularities. Speaking under the first head, he stated that the "bite" held an important place as a factor in the production of approximal decay, for owing to its action, food became wedged into approximal spaces, and by decomposition, promoted decay. Sometimes the very beginning of decay was due to this cause, and even in the larger number of cases where the actual inception of disease occurred before there was any interspace to admit of the lodgment of food, yet when once the coronal wall of the cavity gave way, allowing food to enter in appreciable quantity, the rate of decay was generally much accelerated, and its ravages made to extend rapidly over the whole approximal surface. In those instances where the lodgment of food was the primary cause of decay, the space which admitted the food was due to one of three things, either to simple want of contact in two neighboring teeth otherwise normal, or to a flat instead of a contour filling having been employed on one or both sides of the space, or to the injudicious use of contour instead of flat fillings. Much might be done in the way of operative interference to limit or prevent the action of this predisposing cause to caries, by contour fillings on the one hand, and judiciously shaped spaces on the other; the use of both methods being demanded by the varying conditions met with.

In dealing with approximal cavities, one's aim must be as far as possible to achieve three things, viz:—(1.) To prevent the recurrence of decay. (2.) To render the tooth under treatment useful in mastication. (3.) To protect the gum from irritation. To the question, in what circumstances did contour filling accomplish these things better than any other method? the answer was simple, viz.: when by contouring the filling could be brought into *close* contact with the adjoining tooth. Apart from the question of appearance, the whole value of a contour filling lay in its knuckling up to the neighboring tooth so as to entirely prevent the lodgment of food between the two neighbors, this lodgment being the fruitful source of recurrence of decay, irritation of the gums, and inability to properly masticate. Contour filling was called for then, when the tooth to be treated was standing so close to its neighbor that a reasonable amount of contouring would restore the normal relation of the two. This could be done in the majority of instances, but the minority consisted of not a few cases in which the diseased tooth stood so apart from its neighbor that a restoration of its normal contour did *not* restore the normal relation of the two teeth. In these circumstances, contouring was bad practice, and recourse must be had to flat fillings and judicious shaping of surfaces forming the mesial and distal walls of the space. Broadly speaking, this shaping consisted in making the walls boldly divergent, and very smooth and straight. Sometimes, in order to render the space as non-retentive, and at the same time sacrifice as little tooth substance as possible, the original space might be deliberately increased by means of gutta-percha or wool and mastic before finally shaping and filling. As to the value of such spaces, it could not be denied that, properly made and used only where contouring was inadmissible, they conduced to the comfort and safety of both teeth and gums. It could not however be too strongly maintained that between full contour and wide space there was no middle course, though which to adopt was by no means easy in every case to determine. Desiring to speak without bias, Mr. Matheson was inclined to say, "When in doubt play contour."

It did not infrequently happen that one had to deal with teeth, the relation of which to their neighbor and to the bite necessitated their restoration in contour, but in which disease was so very extensive as to render the wisdom of contour filling questionable. In such cases, the use of all gold collar crowns was invaluable, or in the case of bicuspids, all gold, faced with porcelain. The particular advantage of this form of crown lay in the fact that it could be contoured out in any direction or to any extent, so that it knuckled tightly against the adjoining teeth, and antagonized exactly with its opponents; whilst the collar gave a security and finish which could be obtained by no other method, and produced, if properly adjusted, absolutely *no* irritation of the gum after the day of insertion. Mr. Matheson's own experience of this method had tended to convince him that perfect adaptation of the collar could only be obtained by fitting it to the root in the mouth—he had no faith in the use of a model for fine fitting. As to the modelling of the articulating surface, there was no question in his mind, that the best way was to strike it upon a cast obtained from a wax model, built up and carved for each case, rather than by the use of the die plates sold for the purpose, which made each crown a stereotyped and inartistic copy of every other. Partial gold crowns rendered excellent service where the amount of sound tissue was such as not to warrant its removal for the sake of inserting a complete crown, while at the same time the carious cavity was so far spreading as to make the security and durability of a larger filling quite a question of chances.

Under the second head, Mr. Matheson referred to the periosteal mischief set up by fillings insufficiently cut down to articulate accurately with antagonizing teeth. The inflammatory disturbance varied in intensity; fully pronounced periostitis was rare, more commonly the condition was one of irritation rather than inflammation. A curious point, in many cases, was the sensitiveness to thermal irritants, such as generally characterised irritation of the pulp. Similar symptoms to those named were due, not only to badly finished fillings, but also to a disarrangement in the articulation of one or more teeth produced by the forward pressure of an erupting wisdom tooth. In like manner, the eruption of the second, and even the first permanent molars, in some few instances, produced similar results, as did also the extraction of teeth.

Coming to the last division of his paper, Mr. Matheson limited his remarks to the consideration of the removal of molars or bicuspids for the relief of a general overcrowding of the arch as a whole. Having discussed and defended judicious extraction as against retention of the full number of teeth, he then dealt with the question as to which teeth to remove. Agreeing that the four first molars were the best to get rid of as a rule, he pointed out that many cases, for reasons mainly concerned with the "bite," indicated one of the bicuspids as the tooth to be sacrificed. Bearing in mind the value of the first molar as the chief grinding tooth, the comparative condition of molars and bicuspids ought to be most carefully examined and weighed at the time extraction is decided on. Should the loss of the first molars, after due consideration of all the circumstances, be finally determined on, it was nevertheless generally of the utmost importance that these teeth should be patched up, kept comfortable, and if possible retained until the second molars were quite erupted, and this for three reasons: first, that if the extraction were done at an earlier date the health of the patient would be seriously endangered by inability to masticate properly; second, that the second molars tilt less the later the extraction of the others takes place; and third, that early extraction may injuriously affect the position of the incisors by leaving them and only partially erupted bicuspids to bear all the strain of the "bite."

With reference to "Raising the bite," made necessary in those cases of irregularity where projecting incisors are kept in their abnormal position by the close contact of the lowers, the methods for accomplishing this would vary in detail according to the circumstances of the individual case in hand, but a typical appliance for the purpose, was a simple vulcanite plate made to cover the palate *without* capping any of the teeth. This being worn for some time would prevent the lower incisors from rising any more, their tips being made to impinge against the vulcanite, whilst it allowed of the molars lengthening.

"Jumping" the bite. This unscientific but useful term had been applied to an operation which, when it could be accomplished, was a very pretty one, and one which helped very materially to improve certain mouths. By its means such a sudden alteration is made in the whole articulation, as to justify the use of the word "jumping." Its peculiarity consisted in this, that whilst nearly every other regulating operation aimed simply at altering the position of the teeth in relation to the jaw which carried them, this operation altered the relation existing between the jaws themselves. In other words, the bite is "jumped" by the lower jaw suddenly, in the course of a few days, being made to close upon the upper, in a manner entirely and markedly different to that in which it has been accustomed to close. The operation would be called for in cases where the upper incisors project, where the lowers bite far behind them, and where, besides these conditions, the bicuspids and molars interarticulate abnormally, the lowers closing too far back, by the whole width of a tooth; the chin being consequently very retreating and the whole facial expression weak and foolish. What had to be done here if possible, independently of some retraction of the upper incisors, was an alteration in the position of the lower jaw, so that the chin might be brought bodily forward. This desirable end might be attained by various means. Having referred to a most interesting case communicated by Dr. Bogue and printed in the *Dental Cosmos* for May, 1887, Mr. Matheson mentioned a somewhat similar case recently occurring in his own practice. Having drawn back some prominent upper incisors in a case with a markedly retreating chin, and desiring to keep them in position, he attached a retaining wire to gold caps made to fit the second upper bicuspids—the first molars being gone, and the second scarcely long enough to furnish a secure hold. In order to prevent the bicuspids themselves being brought forward, instead of the incisors being held back, he furnished the caps with tiny inclined planes, directed downwards and backwards for the lower bicuspids to bite against. In a week's time he saw the patient, and was not a little pleased to find that not only had the end he had in view been accomplished, but the lower jaw had come right forward by the width of a tooth, and the retreating chin offended the eye no longer.

It might be observed that in both cases the movement of the lower teeth was due, not to any active mechanical interference with them, but to a spontaneous action on the part of the lower maxilla, when once it was set free from the abnormal position that it was locked in by the bite.—*London Dental Record.*

